# An in vivo assay for osteoclast activity using mouse calvaria

**DOI:** 10.1002/ame2.70112

**Published:** 2025-12-02

**Authors:** Christopher Grieg, Maya Deza Culbertson, J. Patrick O'Connor

**Affiliations:** ^1^ Department of Orthopaedics Rutgers‐New Jersey Medical School Newark New Jersey USA; ^2^ Rutgers‐School of Graduate Studies, Newark Health Sciences Campus Newark New Jersey USA

**Keywords:** bone, mouse model, osteoclast, osteoporosis, resorption assay

## Abstract

Osteoclasts are essential for maintaining healthy bone. Pathological elevation of osteoclastogenesis or osteoclast activity can cause osteoporosis and increase the risk of bone fracture. However, a few options are available for directly measuring osteoclast activity in vivo to test interventions that may affect osteoclasts. Here, we describe an in vivo method to measure osteoclast‐mediated bone loss targeted at normal mouse calvaria. The method employs a novel procedure for measuring osteoclast resorption pits using micro‐computed tomography. The potential utility of this mouse calvaria model to assess therapies targeting osteoclasts was validated using zoledronic acid, which is a nitrogen‐containing bisphosphonate drug used to treat osteoporosis.

## Graphical abstract

This study describes a novel method for measuring osteoclast‐mediated bone resorption in vivo using a mouse model. Localized injection of RANKL in a basement membrane matrix above the cranium induced osteoclast‐mediated bone resorption. Utilizing micro‐computed tomography and a semiautomated three‐dimensional analysis program, osteoclast resorption pit number, pit volume, and total resorbed bone volume were quantified to measure osteoclast activity.

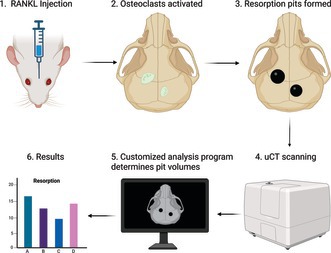

## 1 INTRODUCTION

Bone homeostasis maintains the mechanical strength of living bone and helps provide for calcium homeostasis. Osteoclasts and osteoblasts normally maintain a catabolic and anabolic balance during bone homeostasis. The highly regulated balance between osteoclast‐mediated bone resorption and osteoblast‐mediated bone formation is a critical aspect of bone homeostasis as well as for bone remodeling and bone regeneration.^1^, ^2^ Metabolic bone disorders often stem from a disruption in bone homeostasis. For example, osteoporosis, rheumatoid arthritis, and Padget's disease are linked to overly active osteoclasts.^3^, ^4^ Methods that can measure osteoclast activity in vivo could be useful for identifying new compounds or methods to govern osteoclast activity.

Traditionally, osteoclast resorption assays are performed in vitro. Although in vitro assays can test interventions that affect some aspects of osteoclast activity, they fail to replicate all the factors affecting osteoclasts in living bone.^5^, ^6^, ^7^, ^8^ Available in vivo osteoclast activity assays include the use of radiolabeled compounds that bind to osteoclasts or localize to sites of bone resorption, which can be detected using positron emission tomography or single photon emission computed tomography.^9^, ^10^ Other methods require surgical implantation of materials to induce osteoclast activity or cyclical mechanical loading of rodent limbs to induce bone remodeling.^11^, ^12^ In this article, we present a minimally invasive protocol for inducing osteoclast activity in vivo that is coupled with quantification of the resorbed bone volume using micro‐computed tomography (μCT).

## 2 MATERIALS AND METHODS

### 2.1 Reagents

Matrigel Matrix LDEV‐Free was obtained from Corning (354 234, Corning, NY, USA). GST‐RANKL (receptor activator of nuclear factor kappa‐B ligand) was purified from *Escherichia coli* BL21 transfected with pGEX‐6‐RANKL (a gift from Daved Fremont, Washington University) and purified using glutathione‐Sepharose affinity chromatography (G‐Biosciences, St. Louis, MO, USA) using previously described methods.^13^, ^14^ l(+) tartaric acid and fast red TR salt were obtained from Sigma‐Aldrich (St. Louis, MO, USA). Naphtol AS‐MX phosphate was obtained from Research Products International (Mt Prospect, IL, USA).

### 2.2 Animal models

C57BL/6 mice were obtained from Jackson Laboratory (Bar Harbor, ME, USA).

### 2.3 Matrigel‐RANKL injections

For each injection, 50 μL of Matrigel was mixed with or without 10 μL of GST‐RANKL (1 μg/μL stock) and sufficient phosphate‐buffered saline (PBS) to bring the total volume to 100 μL. For co‐treatment with zoledronic acid (ZA), 1 μL of ZA (5 mmol/L dissolved in water) was added to the RANKL‐Matrigel solution for a final concentration of 50 μmol/L ZA. Mice aged 5, 7, or 10 weeks were anesthetized with 100 μL of ketamine (17.5 mg/mL) and xylazine (2.5 mg/mL) via peritoneal injection before treatment. Each mouse was injected subcutaneously with 100 μL of 50% Matrigel mixture in the cranial surface using a 21‐gauge needle inserted rostrally between the ears to deliver the bolus above the bregma. After 5 days the mice were killed, and their skull was collected.

### 2.4 Cranium collection

Mice were killed by isoflurane inhalation, and the head was removed from the spinal column using sharp scissors. The skin was then incised along the dorsal midline from the nasal bones of the occipital crest, exposing the calvarium. The articulated superior cranial vault was then excised using scissors, carefully debrided of soft tissue, and gently cleaned in PBS. The sample was fixed in neutral buffered formalin for 1 h, washed in water, and stored in 70% ethanol at 4℃ before subsequent staining and scanning.

### 2.5 Tartrate‐resistant acid phosphatase staining

Tartrate‐resistant acid phosphatase (TRAP) was detected as follows. Acetate buffer was prepared using 0.2 mol/L sodium acetate and 50 mmol/L l(+) tartaric acid. TRAP buffer was prepared using acetate buffer with 0.5 mg/mL of Naphtol AS‐MX phosphate and 1.1 mg/mL of fast red TR salt. Crania were placed in 15‐mL conical tubes and submerged in acetate buffer for 20 min at room temperature. The acetate buffer was then aspirated and replaced with TRAP buffer. The samples were incubated at room temperature for 10 min at which point the TRAP buffer was aspirated and the samples were washed with water. After being washed, the samples were dried and images were obtained.

### 2.6 μCT scanning and analysis

Crania were placed in a custom‐designed three‐dimensional (3D) printed fixator (.stl file available at https://github.com/docpoc) in 70% ethanol prior to high‐resolution computerized tomography (μCT) scanning using a Bruker Skyscan 1275 scanner (Micro Photonics Inc., Allentown, PA, USA) with the following settings: 50 kV, 200 μA, 7.5 μm voxel size, frame averaging of 4, and a 0.2° rotation step. Images were reconstructed using Bruker NRecon software (version 1.6.9.8), with a dynamic image range minimum value of 0.009 and a maximum value of 0.06. The 3D reconstructions were loaded into Dragonfly software for Windows (version 2024.1, Comet Technologies Canada Inc., Montreal, Canada). Analysis was performed using a customized routine for Dragonfly developed for this assay (Cranial Osteoclast Resorption Pit Analysis, available at https://github.com/docpoc/Cranial‐Oscteoclast‐Resorption‐Pit‐Analysis; detailed instructions in supplemental files).

### 2.7 Statistical analysis

GraphPad Prism (version 10.4.1, GraphPad Software, Boston, MA, USA) was used for statistical analysis. For μCT resorption analysis, four or six mice (equal number of males and females) per treatment were used. Data are expressed as mean ± standard error. Statistical differences were evaluated using two‐way analysis of variance (ANOVA) followed by Tukey's post hoc corrections and were deemed significant at *p* < 0.05. For sample size analysis, Origin Pro (OriginPro, version 2025, OriginLab Corporation, Northampton, MA, USA) power analysis was utilized.

## 3 RESULTS

### 3.1 Validation of RANKL‐Matrigel injection protocol

**FIGURE 1:**
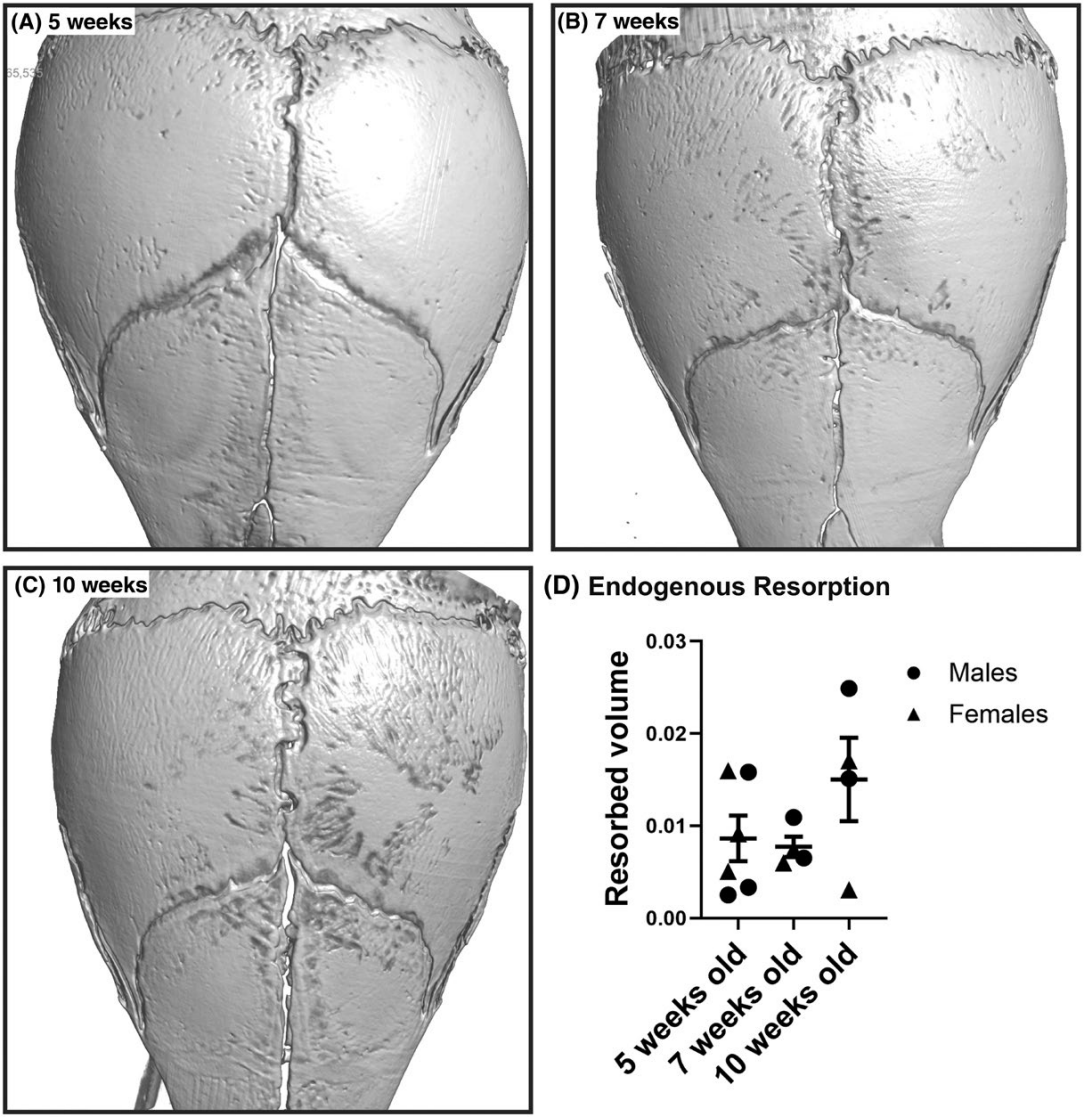
Effect of mouse age on skull morphology. (A–C) Representative images of crania from 5‐, 7‐, and 10‐week‐old nontreated mice. (D) The volume of bone resorbed in the area around the bregma was measured for each age group.

To determine the ideal age of the mice to perform this protocol, calvaria from untreated mice aged 5, 7, and 10 weeks were assessed using μCT (Figure 1A–D). Five‐week‐old mice were chosen based on the relative paucity of apparent bone resorption pits in the parietal and frontal bones (Figure 1).

**FIGURE 2:**
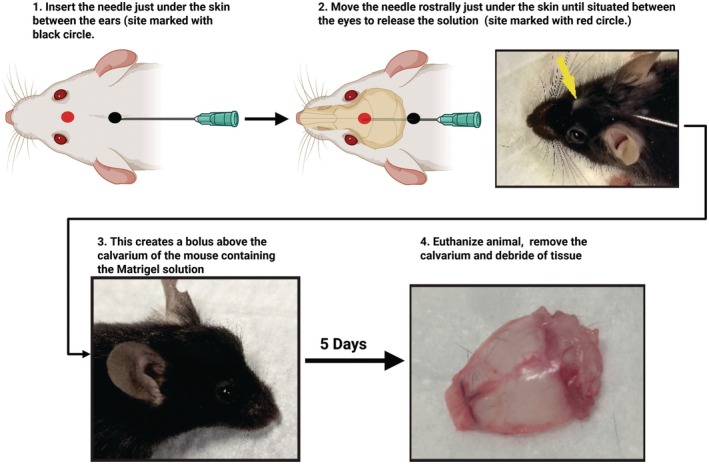
Schematic of animal procedure showing injection of the Matrigel solution above the mouse calvarium and the resected skull.

Matrigel solution with or without RANKL was injected above the parietal and frontal calvaria bones. The injection needle was positioned between the ears and gently inserted rostrally under the skin such that the needle tip was centered just between the eyes to deliver the Matrigel solution above the bregma (Figure 2). The cranium was resected 5 days after injection (Figure 2). Little or no residual Matrigel solution was evident above the skull for either the PBS‐Matrigel‐ or RANKL‐Matrigel‐treated mice by days 3–4.

**FIGURE 3:**
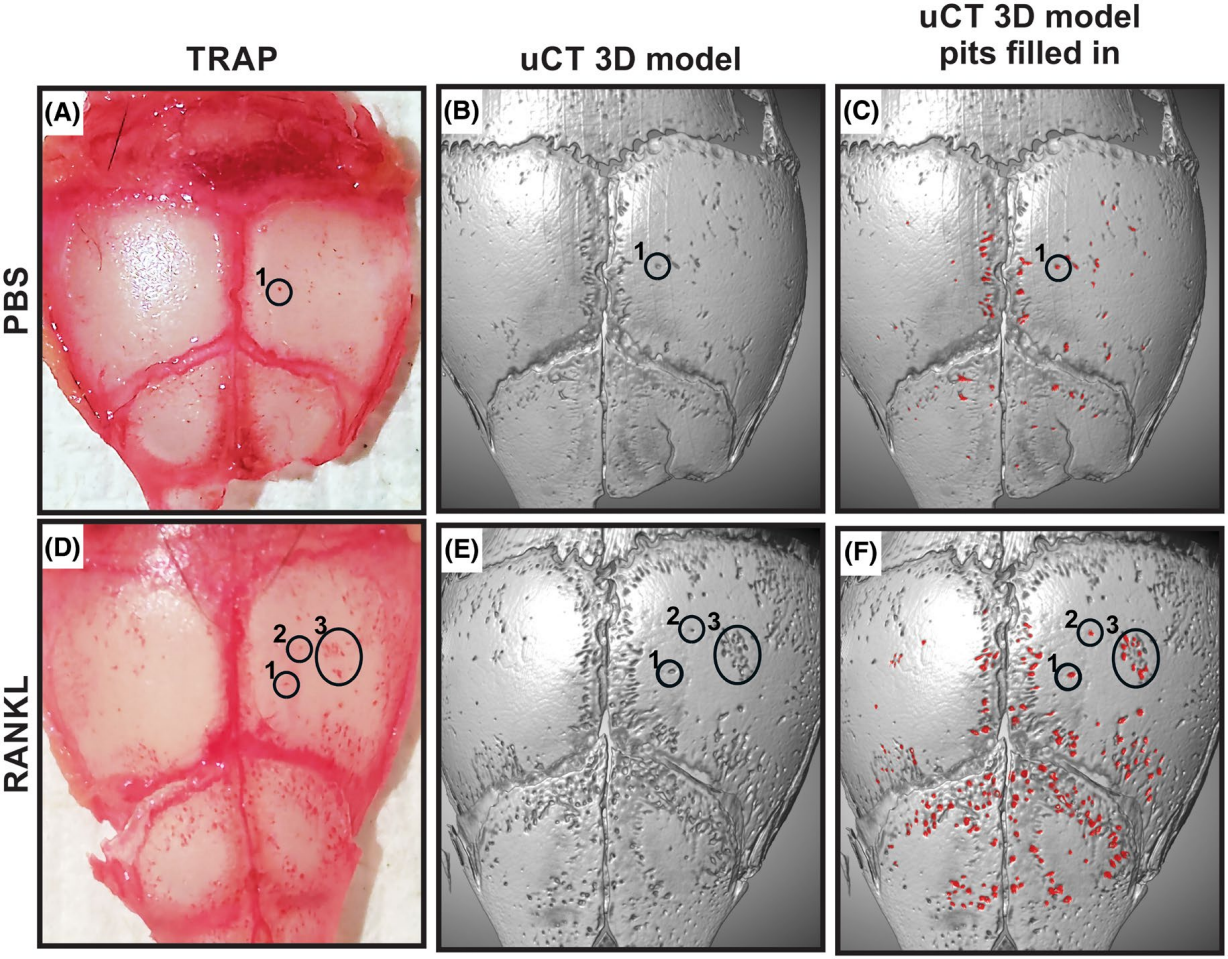
TRAP (tartrate‐resistant acid phosphatase)–stained osteoclast resorption pits co‐localize with resorption pits identified using μCT (micro‐computed tomography). Representative images of TRAP‐stained crania from (A) vehicle control and (D) RANKL‐treated mice, (B and E) their corresponding 3D (three‐dimensional)–generated models from μCT scans, respectively, and (C and F) their corresponding 3D models with filled‐in resorption pits, respectively. TRAP‐stained osteoclasts and their corresponding resorption pits are circled and numbered to demonstrate co‐localization.

To confirm that pits in the calvarial bones identified by μCT were indeed osteoclast resorption pits, resected calvaria from PBS‐Matrigel‐ and RANKL‐Matrigel‐treated mice were stained for TRAP activity, images were obtained, and then μCT scanning was performed (Figure 3). 3D models of the skulls were developed using Dragonfly software (Figure 3B,E). Resorption pits in the 3D models precisely aligned with the TRAP‐stained pits on the cranium (Figure 3A–F). As expected, more μCT pits and TRAP‐stained pits appeared in the calvaria treated with RANKL‐Matrigel.

### 3.2 Validation of Dragonfly program to analyze resorption pits

**FIGURE 4:**
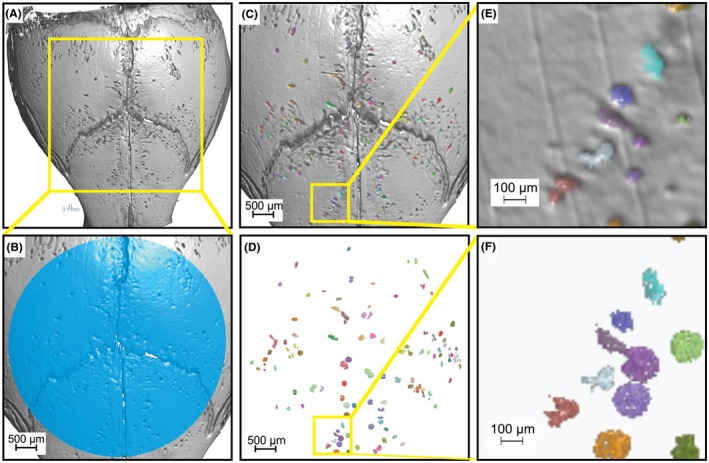
Validation of semiautomated μCT (micro‐computed tomography) data analysis program. Representative images of analysis program steps to identify and measure the resorption pits from the 3D (three‐dimensional) models. (A) The 3D model, (B) the selection of the ROI (region of interest) in blue, (C and E) the pits within the ROI filled, (D and F) and the pits alone.

A program was developed using Dragonfly to analyze the number of pits, the volume of each resorption pit, and the total resorbed volume for a specified region of each calvarium. The program requires the user to center the region of interest (ROI) at the bregma (Figure 4A,B) and enter the optimal thresholding value (Figure S1). For the 5‐week‐old mice, a 7‐mm‐diameter circle was found to be an ideal ROI. The resorption pits within this ROI are then filled and closed, and the volume of the original ROI is subtracted from the closed and filled ROI to isolate the filled renderings of the resorption pits. The program then prompts the user to confirm whether each pit identified by the Dragonfly program on the calvarial surface appears to be a resorption pit or natural porosity that may have been detected by the program (Figure 4C–F). The program subsequently removes any pit volumes marked by the user, leaving just user‐confirmed resorption pits. The Dragonfly program then determines the volume of each user‐confirmed pit as well as the summed pit volume, which is nominally equivalent to the bone resorbed.

### 3.3 RANKL significantly increases resorption

**FIGURE 5:**
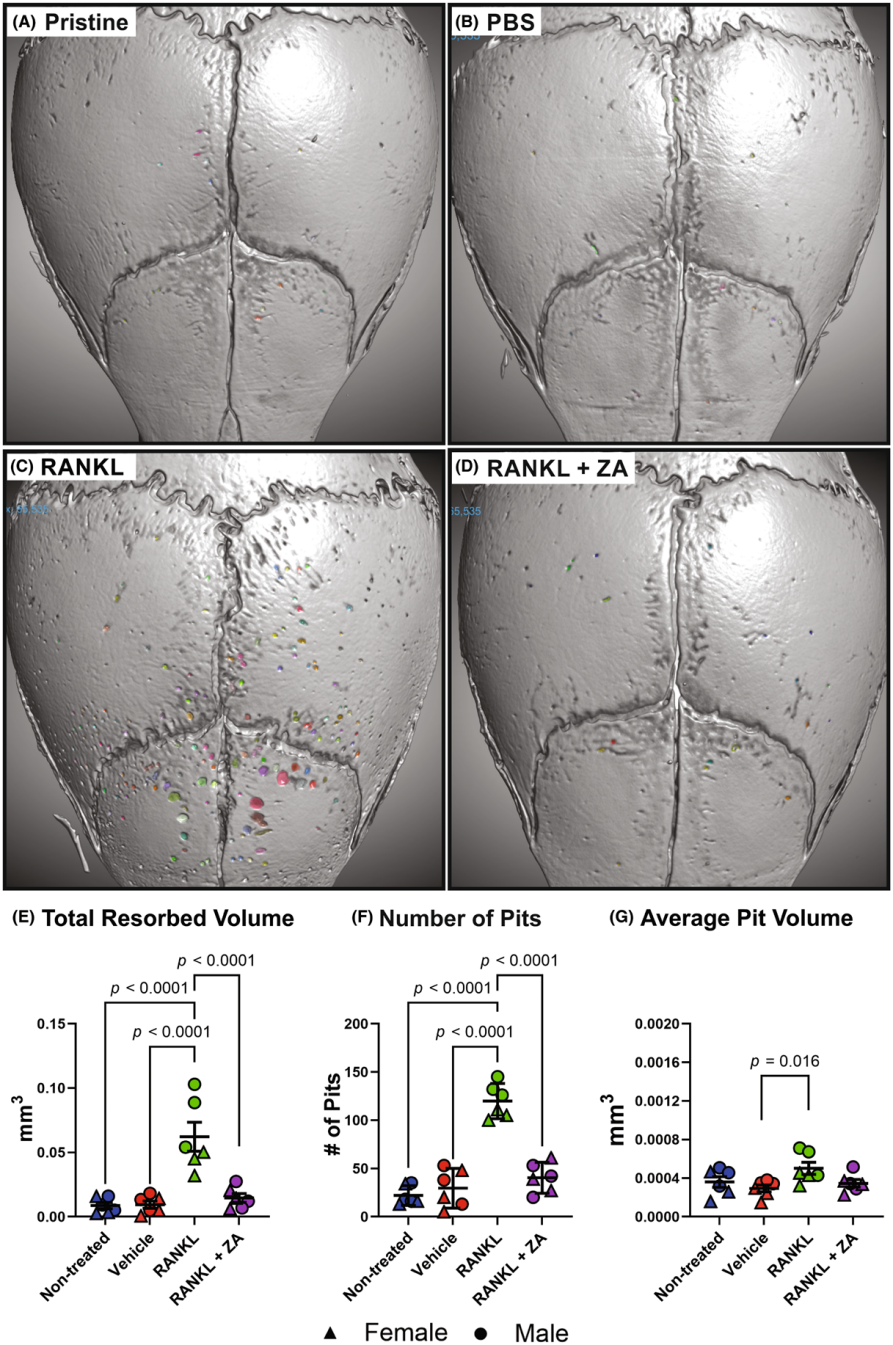
RANKL‐induced pitting is inhibited by zoledronic acid (ZA). Representative images of the 3D (three‐dimensional) models with filled‐in pits for (A) nontreated, (B) vehicle‐treated, (C) RANKL‐treated, and (D) RANKL plus ZA‐treated mice. A significant increase was observed in total resorbed volume (*p* < 0.0001, E), pit number (*p* < 0.0001, F), and average pit volume (*p* = 0.0277, G) for RANKL‐treated samples compared to vehicle‐treated samples. Six (three males and three females) mice per treatment were evaluated using two‐way ANOVA (analysis of variance), and Tukey's post hoc was used to determine significance.

Pristine (uninjected) mice, mice injected with PBS‐Matrigel (vehicle controls), and mice injected with RANKL‐Matrigel (10 μg of GST‐RANKL) were assayed for osteoclast activity (Figure 5A–C). Analysis of the resorption pits showed a significant 6.6‐fold increase in total resorbed volume (*p* ≤ 0.0001) and a 4‐fold increase in resorption pit number (*p* < 0.0001) in the mice treated with RANKL‐Matrigel compared to either the PBS‐Matrigel‐treated mice or the pristine mice (Figure 5E,F; Table 1). The average pit volume in the RANKL‐Matrigel‐treated mice (0.502 μm^3^) was 1.7‐fold greater than that in the PBS‐Matrigel‐treated mice (0.293 μm^3^; *p* = 0.016; Figure 5G; Table 1).

**TABLE 1:** Osteoclast resorption analysis.

	Average number of resorption pits		Average volume per pit (μm^3^ × 1000)		Total resorbed volume (μm^3^ × 1000)	
Treatment effect (two‐way ANOVA)	*p* < 0.0001		*p* = 0.0208		*p* < 0.0001	
Group	Count	*p*‐value versus RANKL	Volume	*p*‐value versus RANKL	Volume	*p*‐value versus RANKL
Pristine	22	**<0.0001**	360	ns	8646	**<0.0001**
PBS	30	**<0.0001**	293	**0.0160**	9368	**<0.0001**
RANKL+ZA	40	**<0.0001**	347	ns	14 570	**<0.0001**
RANKL	120	–	502	–	62 170	–

Abbreviations: ANOVA, analysis of variance; ns, not significant; PBS, phosphate‐buffered saline; ZA, zoledronic acid.

bold values of statistical analysis indicates *p* < 0.05 is significant.

Analysis of all treatment groups using two‐way ANOVA identified a significant difference between the sexes for the total resorbed bone volume (*p* = 0.018) and average pit size (*p* = 0.006), whereas no difference was observed in the number of resorption pits. However, within each treatment group, sexual dimorphism was detected only in the RANKL‐treated samples (total resorbed bone volume: *p* = 0.0007, average pit size: *p* = 0.031; Table 2).

**TABLE 2:** Sexual dimorphism in in vivo osteoclast activity assay.

	Average number of resorption pits			Average volume per pit (μm^3^ × 1000)			Total resorbed volume (μm^3^ × 1000)		
Sex effect (2‐way ANOVA)	*p* = 0.1498			*p* = 0.0310			*p* = 0.0179		
Group	M	F	*p*‐value	M	F	*p*‐value	M	F	*p*‐value
Pristine	23	21	–	427	293	0.139	10 070	7218	0.7657
PBS	35	24	–	358	228	0.150	12 150	6586	0.5631
RANKL	134	105	–	604	400	**0.031**	81 930	42 410	**0.0007**
RANKL+ZA	38	42	–	382	311	0.419	15 450	13 690	0.8541

Abbreviations: ANOVA, analysis of variance; PBS, phosphate‐buffered saline; ZA, zoledronic acid.

bold values of statistical analysis indicates *p* < 0.05 is significant.

### 3.4 Testing osteoclast‐targeting drugs

To determine if drugs affecting osteoclast function caused detectable changes in resorption using this model, mice were treated with RANKL‐Matrigel containing ZA and compared to mice treated with RANKL‐Matrigel only. Addition of ZA significantly reduced RANKL‐induced resorption as evidenced by a significant 76.6% decrease in total resorption volume (*p* < 0.001) and a 66.3% decrease in the number resorption pits (*p* < 0.001) (Figure 5C–F; Table 1).

### 3.5 Sample size

To establish the required sample sizes, the total resorbed volume values for the vehicle and RANKL‐treated samples were compared, and various percentage differences between the means were examined for a power of 0.8 and an α value of 0.05. Required sample sizes of 33, 10, and 6 were found for 25%, 50%, and 75% effect size, respectively. The required sample sizes for the number of resorption pits were found to be 11, 5, and 4 for 25, 50, and 75 percentage effect sizes, respectively (Table 3).

**TABLE 3:** Sample sizes.

Sample sizes required to reach significance for various percentage differences		
Percentage difference between vehicle and RANKL means (%)	Group size (total resorbed volume)	Group size (number of pits)
25	33	11
50	10	5
75	6	4
100	5	3

## 4 DISCUSSION

We described an effective assay for quantifying osteoclast resorption in vivo. Locally applying RANKL in Matrigel above the cranium of 5‐week‐old mice is a minimally invasive but effective procedure to induce quantifiable osteoclast resorption. We selected 5‐week‐old mice for use in this assay because at that point, the skull has yet to undergo significant remodeling and exhibits a relative smooth surface with a few resorption pits. As mice age, skull growth and remodeling increases the baseline number of resorption pits, whereas increased vascularization causes corrugations in the bone.^15^ Thus, distinguishing induced resorption pits from natural remodeling in skulls from mice aged >7 weeks would become more difficult (Figure 2).

We found a significant increase in TRAP‐stained resorption pits on the outer skull of the RANKL‐Matrigel‐treated mice, demonstrating a clear increase in osteoclast activity. The source of the osteoclasts induced by RANKL is not clear. The induced osteoclasts could arise from local macrophage or dendritic cell populations, could migrate from the sagittal and coronal sutures where bone formation occurs, or may arise from systemic sources.^16^, ^17^, ^18^, ^19^ By comparing the TRAP‐stained pits to resorption pits identified using μCT imaging and analyses, a clear parallel between the two was observed (Figure 3). Subsequently, the resorption pits identified using μCT were used to quantify osteoclast activity. To facilitate measuring osteoclast activity from μCT imaging, we developed a program in Dragonfly software that provides for a consistent, semiautomated analysis of the reconstructed μCT image data to measure the number of resorption pits, average pit volume, and the total resorbed bone volume (Figure 4). We show that when RANKL is administered, a significant increase in resorption occurs compared to pristine or PBS‐treated mice. Conversely, simultaneous treatment with RANKL and ZA led to significantly fewer resorption pits and a decrease in total resorbed volume. The experimental results demonstrate the utility of this assay to quantify osteoclast activity in vivo.

The described in vivo assay for osteoclast activity offers two significant advantages over other in vivo assay methods. Previously described methods used RANKL to induce osteoclast activity in vivo by surgical implantation of RANKL absorbed into carriers, such as collagen sponge.^12^, ^20^, ^21^ The use of Matrigel to localize the delivery of RANKL simplifies this process, is less invasive to mice, and provides a highly repeatable method for delivering RANKL. Another significant advantage is the semiautomated analysis of the μCT imaging data that provide for a rigorous, quantitative outcome of osteoclast activity.

Based on the variation observed in osteoclast activity between the PBS‐treated and RANKL‐treated mice, this assay procedure can practically detect 50% differences in vivo for RANKL‐induced osteoclast activity using a reasonable number of experimental animals (6–12), depending on the desired primary outcome (osteoclast resorption pits or bone resorbed; Table 3). The utility of this assay should be easily extended to assessing genetically modified mouse models or testing the acute effects of systemic or local therapeutics on osteoclast activity (Figure 5).

Even though the mice used in the study were juveniles, we found significant differences in average pit volume and total bone resorbed volume between RANKL‐treated male and female mice (Table 2). Interestingly, this difference is due to a larger resorbed volume and pit size in males compared to females. In contrast, previous studies found that bone marrow cell cultures from adult female mice typically produce more osteoclasts with more resorptive activity than bone marrow cell cultures from male mice.^22^ However, Zanotti et al.^23^ found no difference in osteoclastogenesis when bone marrow cells from 1‐month‐old male and female C57BL/6 mice were used. Similarly, Abe and Aoki found no differences in femur osteoclasts or resorption pits between 1‐month‐old male and female mice but did observe larger resorption areas in 7‐ and 14‐week‐old female mice.^24^ One potential cause of this could be the increased production of estrogen in the female mice. Estrogen both inhibits osteoclast activity and leads to osteoclast apoptosis.^25^, ^26^ In C57BL/6 female mice, serum estradiol significantly increases between 4 and 5 weeks of age.^27^, ^28^ Because estradiol peaks in the female mice directly at the time point used for this study, the high serum estradiol likely accounts for the observed sexual dimorphism. For future studies, using 7‐week‐old female mice rather than 5‐week‐old female mice may be beneficial. Further investigation is needed to determine if elevated estrogen levels in juvenile female mice are the cause of the observed sexual dimorphism.

Other approaches to measure osteoclast activity utilize histomorphometric or μCT analyses of parameters such as bone volume or porosity. Histomorphometry relies on imaging and 2D analysis of thin bone sections. Although a powerful tool, this method has inherent limitations. The analysis is limited to only the resorption observed in the slices examined, making it likely that many resorption pits will be only partially captured in the section or missed altogether. Moreover, this method focuses only on resorption in the two‐dimensional plane, which does not account for the volume of the resorption pit. Comparing μCT analysis of bone volume or porosity between samples does not exclude the natural variation in bone between samples. The present protocol addresses these concerns by using a 3D model that accounts for all the resorption pits in the ROI and measures the volume of the pit, not just an area. Furthermore, by examining the induced resorption pits, natural bone variation between samples is eliminated.

Although this study demonstrates the utility of this in vivo approach to measure osteoclast activity, additional experiments to modify the procedure for specific cases may be needed. For instance, only one dose of RANKL (10 μg) was used in this study. RANKL dosing would be expected to alter the osteoclast response, so a minimum or maximum‐effective dose may be more useful in other situations. Similarly, osteoclast activity was measured only at a single time point (5 days) after RANKL administration. Should larger effect sizes be needed, extending the time after RANKL dosing may be useful. Although the present results showed a clear antiosteoclast effect of ZA, we did not attempt to use other local or systemic antiresorptive therapies nor did we perform a dose–response analysis of locally applied ZA on osteoclast activity. Because osteoclastogenesis in this assay was induced by a localized bolus of RANKL, either testing of local or systemic anti‐RANKL antibody therapies would need to overcome the initial bolus dose of RANKL, or the anti‐RANKL therapy would need to be initiated once the bolus dose has dissipated. As shown by Kuritani et al.^29^ local treatment of mouse calvaria with lipopolysaccharide can elicit an inflammation response and osteoclastogenesis, which could be an alternative approach for inducing osteoclastogenesis when anti‐RANKL therapies are tested. We also did not attempt to alter osteoclastogenesis at the target site by inclusion of cells, growth factors, cytokines, or other agents by coadministration with RANKL‐Matrigel. Optimizing such approaches will most likely be agent specific.

This study establishes the validity of this assay to measure osteoclast activity in vivo and the utility of the described μCT methods for quantifying osteoclast activity. We expect that this approach will aid others investigating osteoclast biology and the effects of mutations, drugs, cytokines, growth factors, or cells on osteoclast function.

## AUTHOR CONTRIBUTIONS

**Christopher Grieg:** Conceptualization; data curation; formal analysis; investigation; methodology; validation; visualization; writing – original draft; writing – review and editing. **Maya Deza Culbertson:** Conceptualization; resources; writing – review and editing. **J. Patrick O'Connor:** Conceptualization; funding acquisition; methodology; project administration; resources; supervision; writing – original draft; writing – review and editing.

## FUNDING INFORMATION

This research was supported by the National Institute of Arthritis and Musculoskeletal and Skin Diseases of the National Institutes of Health (grant R01AR069044); the Rutgers‐New Jersey Medical School Department of Orthopedics.

## CONFLICT OF INTEREST STATEMENT

The authors have no conflicts of interest.

## ETHICS STATEMENT

All animal procedures were approved by the Rutgers Institutional Animal Care and Use Committee (protocol 201800006).

## PATIENT CONSENT

Not applicable.

## PERMISSION TO REPRODUCE MATERIAL FROM OTHER SOURCES

Not applicable.

## CLINICAL TRIAL REGISTRATION

Not applicable.

## Supporting information


**Figure S1.** Optimal thresholding. Representative images to determine optimal thresholding (thresholding is in red over white bone). (A) Image of a thresholding value that is too low and does not define the pits. (B) Image of a thresholding value that is too high and does not pick up all the bone. (C) Image of optimal threshold value in which the bone is all picked up and the pits are well defined.

## Data Availability

Primary data is available upon reasonable request.
