# Supplementary figures and images for: An in vivo assay for osteoclast activity using mouse calvaria
Source: Animal Model Exp Med. 2025 Dec 2;9(1):41–9. doi: 10.1002/ame2.70112 (PMC12907971; doi:10.1002/ame2.70112)

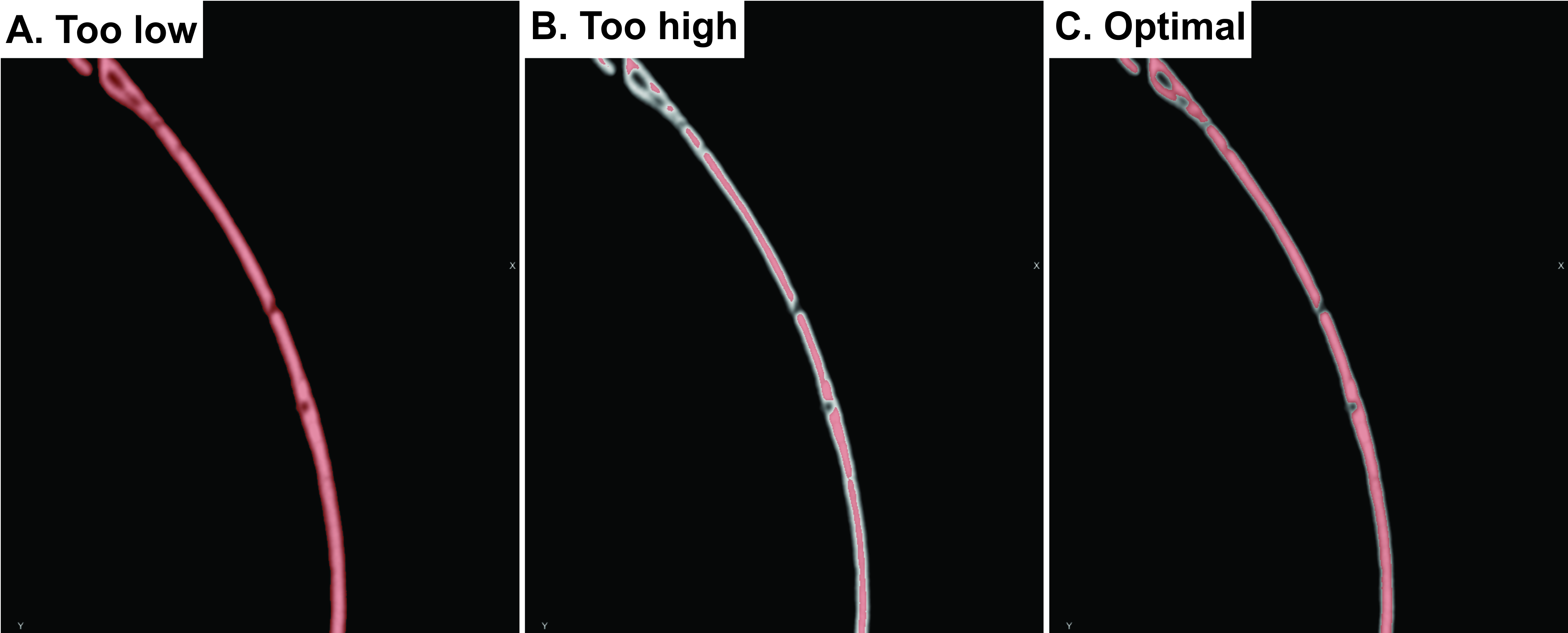

Supplement: Supplementary file 1 — Figure S1. Optimal thresholding. Representative images to determine optimal thresholding (thresholding is in red over white bone). (A) Image of a thresholding value that is too low and does not define the pits. (B) Image of a thresholding value that is too high and does not pick up all the bone. (C) Image of optimal threshold value in which the bone is all picked up and the pits are well defined. [file AME2-9-41-s001.tif]
